# Characterization of the polyphenol oxidase gene family reveals a novel microRNA involved in posttranscriptional regulation of *PPOs* in *Salvia miltiorrhiza*

**DOI:** 10.1038/srep44622

**Published:** 2017-03-17

**Authors:** Caili Li, Dongqiao Li, Jiang Li, Fenjuan Shao, Shanfa Lu

**Affiliations:** 1 Institute of Medicinal Plant Development, Chinese Academy of Medical Sciences & Peking Union Medical College, No. 151, Malianwa North Road, Haidian District, Beijing 100193, China

## Abstract

*Salvia miltiorrhiza* is a well-known material of traditional Chinese medicine. Understanding the regulatory mechanisms of phenolic acid biosynthesis and metabolism are important for *S. miltiorrhiza* quality improvement. We report here that *S. miltiorrhiza* contains 19 polyphenol oxidases (*PPOs*), forming the largest *PPO* gene family in plant species to our knowledge. Analysis of gene structures and sequence features revealed the conservation and divergence of *SmPPOs. SmPPOs* were differentially expressed in plant tissues and eight of them were predominantly expressed in phloem and xylem, indicating that some *SmPPOs* are functionally redundant, whereas the others are associated with different physiological processes. Expression patterns of eighteen *SmPPOs* were significantly altered under MeJA treatment, and twelve were yeast extract and Ag^+^-responsive, suggesting the majority of *SmPPOs* are stress-responsive. Analysis of high-throughput small RNA sequences and degradome data showed that miR1444-mediated regulation of *PPOs* existing in *P. trichocarpa* is absent from *S. miltiorrhiza*. Instead, a subset of *SmPPOs* was posttranscriptionally regulated by a novel miRNA, termed Smi-miR12112. It indicates the specificity and significance of miRNA-mediated regulation of *PPOs*. The results shed light on the regulation of *SmPPO* expression and suggest the complexity of SmPPO-associated phenolic acid biosynthesis and metabolism.

Polyphenol oxidases (PPOs) are copper-binding enzymes widely distributed in plants. They utilize oxygen to catalyze exclusively the dehydrogenation of catechols (i.e., *o*-diphenols) to the corresponding *o*-quinones (known as the diphenol oxidase/catecholase/*o*-diphenolase activity; EC 1.10.3.1) or act as bifunctional enzymes to catalyze the oxidation of monophenols to *o*-diphenol intermediates (known as the tyrosinase/cresolase/monophenolase activity; EC 1.14.18.1) and the subsequent oxidation of *o*-diphenols to the corresponding *o*-quinones^1^. The highly reactive *o*-quinones can then polymerize with themselves or react with functional groups of proteins to produce black, brown or red pigments. It is the major cause of fruit browning and discoloration of plants during processing^2^.

Postharvest browning of fruit and vegetables may cause nutritional loss and has a negative effect on consumer acceptability^3^. On the other hand, the brown color developed by PPOs is of great importance in the quality of black tea and cacao^4^. Due to the economic significance, studies on plant PPOs have mainly concentrated on their roles in postharvest discoloration of plants and on preventing PPO-mediated browning reactions. Although it has been over a century of intensive research on PPOs, the physiological function of PPOs in plants is still not well understood^5^.

Accumulating evidence suggests that PPOs are involved in plant response to biotic and abiotic stresses^5^. Transgenic tomato with reduced PPO activity showed high drought resistance than wild-type and PPO-over-expressed plants^6^. PPOs in *Populus* are Cu- and Zn-responsive and involved in metal ion-associated gene networks^7^. Over-expression of a potato PPO cDNA in tomato resulted in decreased susceptibility to *Pseudomonas syringae* pv. Tomato^8^, while down-regulation of PPOs in tomato and dandelion led to enhanced disease susceptibility^6^,^9^. Forest tent caterpillar larvae reared on transgenic hybrid aspen foliage over-expressing *PPO* genes showed decreased growth rates^10^. Consistently, negative correlation between PPO activity and larvae consumption and growth was observed when cotton bollworm, beet armyworm and common cutworm were fed on *PPO*-transgenic tomato plants^11^,^12^, and jasmonate-dependent induction of PPO activity in tomato foliage was found to be important for defense against beet armyworm^13^. Additionally, PPOs are involved in seeds against herbivores, bacteria and fungi and proposed to be vital for seed survival in the soil^14^. In addition to being stress-responsive, a subset of *PPOs* is targeted by miR1444 for direct cleavage in *P. trichocarpa*^15^. MiR1444-mediated posttranscriptional regulation of *PPOs* plays significant roles in copper homeostasis and stress responses^7^,^15^. However, miR1444 exists only in Salicaceae plants. The regulatory role of miRNAs in *PPOs* from other plant species is largely unknown.

Although PPOs appear to be vital in plants, the roles of PPOs played may vary among species. It is attributed to the diversity of PPO sequences^5^. A plant PPO protein typically consists of three domains: an N-terminal targeting signal, a dicopper centre, and a C-terminal region^16^. The N-terminal targeting signal is usually a chloroplast transit peptide (cTP) responsible for the translocation of PPO proteins to the thylakoid lumen^1^; while in *Antirrhinum majus* aureusidin synthase 1 (AmAS1) and *Populus trichocarpa* PtrPPO13, it is a secretory pathway signal peptide (SP) directing PPO proteins to vacuola^1^,^17^. The dicopper centre includes two copper-binding domains, CuA and CuB, each of which is approximately 50 amino acids in length^16^. Sequence comparison suggests that CuA is more variable than CuB and this variation may affect substrate preferences^16^. The C-terminal region consists of a 50 amino acid PPO1_DWL domain and a 140–150 amino acid PPO1_KFDV domain^16^. The functional significance of this region is not known, but it is susceptible to proteolytic cleavage in bean and sugarcane^16^.

*Salvia miltiorrhiza* Bunge is a well-known material of various traditional Chinese medicines (TCMs) widely used in the treatment of cardiovascular, hyperlipidemia, cerebrovascular and acute ischemic stroke diseases^18^. It is also an emerging model system for medicinal plant biology^19^. Hydrophilic phenolic acids are a group of the main active pharmaceutical ingredients of *S. miltiorrhiza*. Understanding the regulatory mechanisms of phenolic acid biosynthesis and metabolism are important for *S. miltiorrhiza* quality improvement^20^. Although the significance of *PPOs* has been shown in various plants, no information is available for *PPOs* in *S. miltiorrhiza*. Here, we report genome-wide prediction, molecular cloning and expression analysis of 19*S. miltiorrhiza SmPPO* genes. We show the conservation and divergence of *SmPPOs* in gene structures, sequence features and expression patterns. We found that the majority of *SmPPOs* were stress-responsive and a novel miRNA was involved in posttranscriptional regulation of *SmPPOs*. The results shed light on the regulation of *SmPPO* expression and suggest the complexity of PPO-related phenolic acid biosynthesis and metabolism in *S. miltiorrhiza*.

## Results

### Prediction and molecular cloning of *S. miltiorrhiza SmPPO* genes

Through genome-wide computational search and subsequent gene model prediction, a total of 12 full-length and 7 partial *SmPPOs* were obtained. To verify the prediction and get full-length coding sequences (CDSs) of all 19 *SmPPOs*, molecular cloning approaches, including 5′ and 3′ RACE and PCR amplification of coding regions, were performed. The identified *SmPPOs* were named *SmPPO1*–*SmPPO19*, respectively. The deduced SmPPO proteins have amino acid numbers from 564 to 625, isoelectric points (p*I*) from 5.23 to 8.60, and molecular weights (Mw) from 49.0 to 66.0 kDa. All of the cloned *SmPPO* CDSs have been submitted to GenBank under the accession numbers shown in Table 1.

### Phylogenetic analysis of SmPPOs and PPOs from other plant species

**Figure 1:**
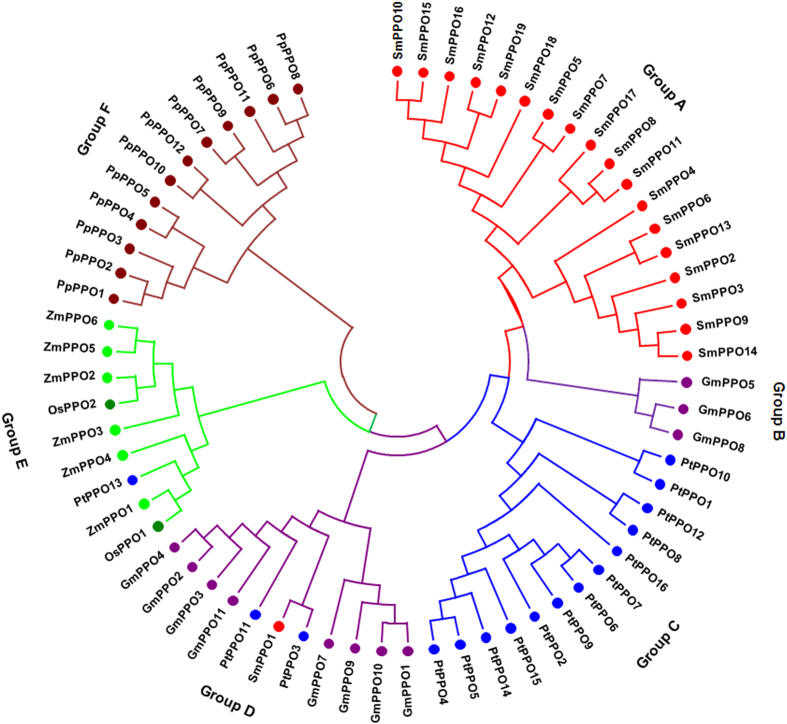
Phylogenetic relationship of PPO proteins in six plant species. The phylogenetic tree was built using the neighbor-joining (NJ) method implemented in MEGA7.0 with 1000 bootstrap replicates. PPOs from *S. miltiorrhiza* (Sm), *P. trichocarpa* (Pt), *Glycine max* (Gm), *Oryza sativa* (Os), *Zea mays* (Zm) and *Phycomitrella patens* (Pp) are indicated by bullet points with different colors.

To examine the phylogenetic relationship among 70 PPO proteins from *S. miltiorrhiza, P. trichocarpa, Physcomitrella patens*, rice (*Oryza sativa*), maize(*Zea mays*) and soybean (*Glycine max*), an unrooted Neighbor-Joining (NJ) tree was constructed (Fig. 1). PPO proteins can be divided into six subgroups (Fig. 1). Phylogenetic analysis reveals species-specific PPO subgroups, which is consistent with previous observation^16^. In *P. patens, S. miltiorrhiza* and *P. trichocarpa*, PPO expansion is largely a consequence of lineage-specific gene duplication and subsequent divergence. 12 PPO sequences from *P. patens* are clustered into a group. 18 of 19 SmPPOs belong to single group. 14 PtPPOs form a monophyletic group. It indicates that the expansion and diversification of these PPOs was occurred independently in different lineage. OsPPO1, OsPPO2 and PtPPO13 are clustered with ZmPPOs in group E, whereas PtPPO3, PtPPO11 and SmPPO1 are grouped with GmPPOs in group D. It indicates that these PPOs from different plant species share common ancestors.

In total, 17 *SmPPO* genes belonging to six paralogous groups were identified from the 19 cloned *SmPPO* genes (Table 2). In order to examine the divergence of these paralogs, Ka and Ks were calculated for open reading frames (ORFs) and coding sequences of CuA domain, CuB domain, DWL domain, KFDV domain (Supplementary Fig. S1). On average, the *Ka* values of ORF, CuA domain, CuB domain, DWL domain, KFDV domain were 0.29, 0.15, 0.23, 0.29, and 0.26, respectively. The *Ka* values were not significantly different, except for the CuA domain, as revealed by the t-test (*P* < 0.05). The *Ks* values from ORF, CuA domain, CuB domain, DWL domain, and KFDV domain were similar (0.75, 0.60, 0.65, 0.70 and 0.64, respectively). The values of *Ka* and *Ks* suggest that *SmPPOs* are highly conserved. The average *Ka*/*Ks* ratio of CuA domain (0.25) is much smaller than the average *Ka*/*Ks* ratio of other four sources (*Ks* values of ORF, CuB domain, DWL domain, KFDV domain were 0.38, 0.35, 0.41 and 0.40, respectively). The Ka/Ks ratios indicate strong purifying selection on SmPPOs, and the CuA domain is more conserved than CuB domain.

### Conservation and divergence of *SmPPO* gene structures, conserved domains and motifs

**Figure 2:**
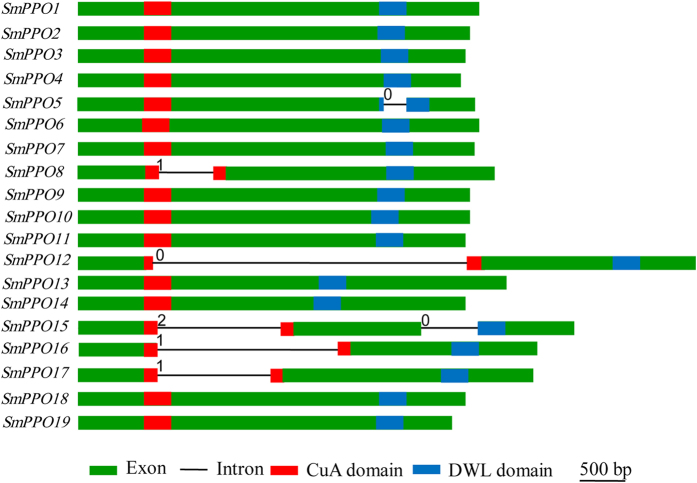
Intron-exon structures of *SmPPO* genes. Red boxes, blue boxes, green boxes and lines indicate CuA domain, PPO_DWL domain, exons and introns, respectively. The numbers indicate intron phases.

Gene structure analysis showed that the intron number in the coding regions of 19 *SmPPO* genes varied between 0 and 2 with the majority (63%) to be 0 (Fig. 2). Consistently, most *PtPPO* genes have no introns in the coding regions^16^. Only a few eudicot PPOs contain introns, such as the cherimoya *PPO* gene^16^ and four Salicaceae *PPOs*^21^. It suggests the similarity of *PPOs* in gene structures. Further examining the position of introns showed that *SmPPO8, SmPPO12, SmPPO16* and *SmPPO17* contained an intron located in the region encoding CuA domain. *SmPPO5* has an intron in the PPO_DWL domain-encoding region. *SmPPO15* contains two introns. One is located in the CuA domain-encoding region, whereas the other one is located in the PPO_DWL domain-encoding region (Fig. 2).

**Figure 3:**
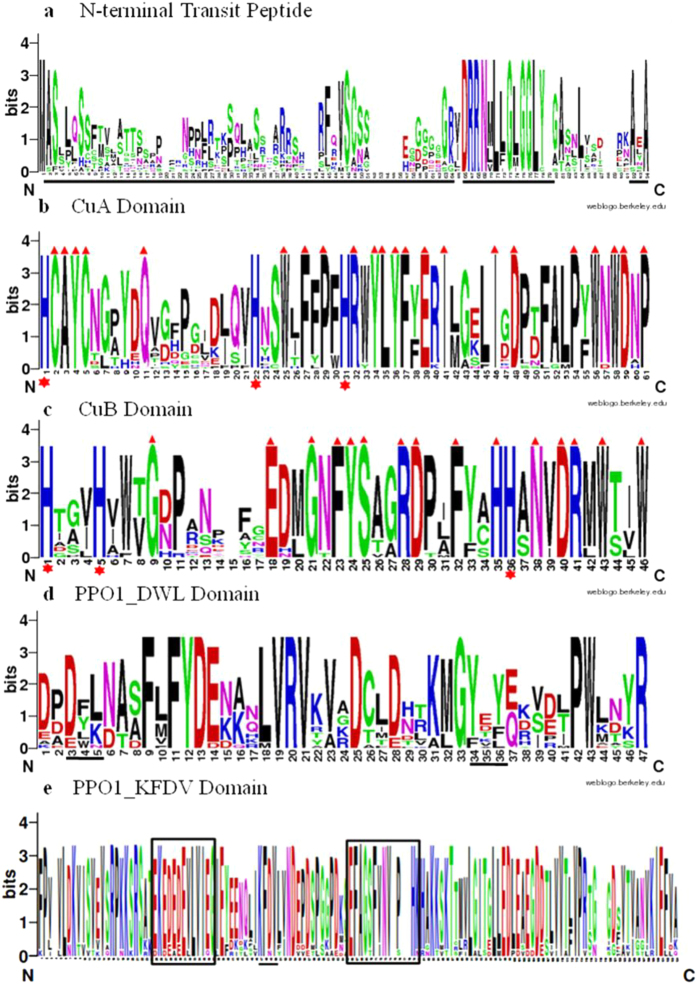
Highly conserved domains in SmPPO proteins. (**a**) The N-terminal transit peptide. The transit peptide sequences are underlined. (**b**) The CuA domain. Red triangle indicates absolutely conserved residues. Red stars indicate three conserved histidine residues. (**c**) The CuB domain. Absolutely conserved residues and conserved histidine residues are indicated by red triangle and stars, respectively. (**d**) The PPO1_DWL domain. The DWL motif and the tyrosine (YxY) motif are underlined. (**e**) The PPO1_KFDV domain. The thylakoid transfer domain, the KFDV motif and the alanine (AxA) cleavage motif are underlined. The conserved EEEEEVLVI and EFAGSF motifs are boxed.

Plant PPO proteins typically consist of three domains: an N-terminal targeting signal, a dicopper centre, and a C-terminal region^16^. These domains are highly conserved in plants. In order to elucidate sequence features of domains and the degree of conservation of each residue, multiple sequence alignment was performed and sequence logos for these domains of SmPPOs were created using WebLogo. The results showed that the distribution of residues in domains of SmPPOs was quite similar to other plant PPOs^16^. The N-terminal region of SmPPOs, varying from 80 to 90 amino acids (Fig. 3a), harbors the chloroplast transit peptide (cTP) (Table 3). The cTP (～60 aa) is rich in serine residues. All chloroplast transit peptides of SmPPOs display conserved motifs for a thylakoid transfer domain (TTD) and an alanine cleavage motif (AxA) (Fig. 3a). The dicopper centre includes two copper-binding domains, CuA and CuB (Fig. 3b,c), each of which is approximately 50 amino acids in length^16^. The highly conserved histidine residues were found in CuA and CuB domains. The CuA domain of SmPPOs contains a highly conserved HCAYC motif (HCAYC) (Fig. 3b), which also exists in other plant PPOs^16^. The second Cys in this motif is predicted to form a thioether bond with the second conserved histidine of the CuA domain. The sequences between the HxxxC motif and the second conserved histidine are highly variable. In addition to the three histidines, several other amino acids located downstream of the third conserved histidine in the CuA domain are also conserved. It includes arginine, tyrosine, leucine, phenylalanine, glutamic acid, aspartic acid, proline, and tryptophan. In the CuB domain, there are two conserved histidine residues (HxxxH motif) (Fig. 3c). The CuB domain contains several highly conserved residues (Fig. 3c). Sequence comparison suggests that CuB is more variable than CuA in SmPPOs (Fig. 3b,c). The C-terminal portion of SmPPOs shows highly conserved features. It has the PPO1_DWL domain and the PPO1_KFDV domain (Fig. 3d,e)^16^. PPO1_DWL domain is approximately of 50 amino acids in length (Fig. 3d). It contains a conserved DWL sequence motif and a tyrosine motif (YxY) (Fig. 3d). The PPO1_KFDV domain has a highly conserved sequence motif, KFDV (Fig. 3e). Additionally, the EEEEEVLVI motif enriched in glutamic acid residues and the EFAGSF motif are present in many SmPPOs (Fig. 3e) and other land plant PPOs^16^. The functional importance of these domains and motifs remains to be elucidated.

### Expression profiles of *SmPPO* genes in *S. miltiorrhiza*

**Figure 4:**
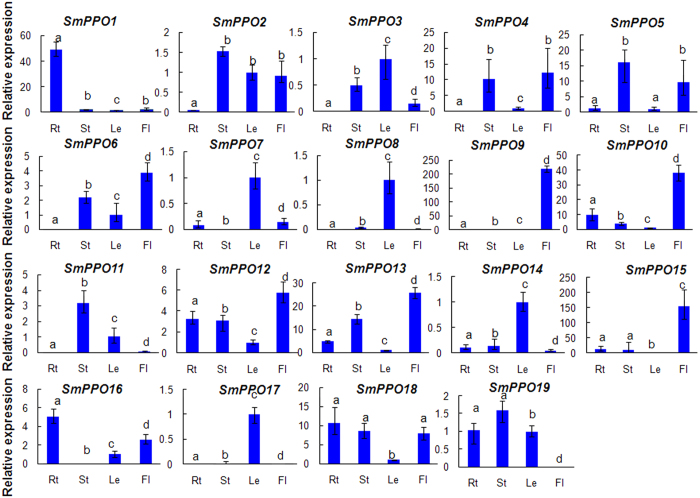
Expression of *SmPPOs* in roots (Rt), stems (St), leaves (Le) and flowers (Fl) of *S. miltiorrhiza*. The expression levels were quantified using the quantitative RT-PCR method. Transcript levels in leaves were arbitrarily set to 1 and the levels in other tissues were given relative to this. Error bars represent standard deviations of mean value from three biological and three technical replicates. ANOVA (analysis of variance) was calculated using SPSS. *P* < 0.05 was considered statistically significant.

In order to preliminarily elucidate the physiological roles of *SmPPOs*, we detected the levels of *SmPPO* transcripts in roots, stems, leaves and flowers of *S. miltiorrhiza* plants. Differential expression was observed (Fig. 4). *SmPPO1* and *SmPPO16* were predominantly expressed in roots. *SmPPO11* was predominantly expressed in stems. *SmPPO3, SmPPO7, SmPPO8, SmPPO14* and *SmPPO17* were strongly expressed in leaves. *SmPPO6, SmPPO9, SmPPO10, SmPPO12, SmPPO13* and *SmPPO15* exhibited the highest expression in flowers. The other 5 *SmPPOs* were mainly expressed in at least two tissues analyzed. Differentially expressed *SmPPOs* may be involved in different physiological processes.

**Figure 5:**
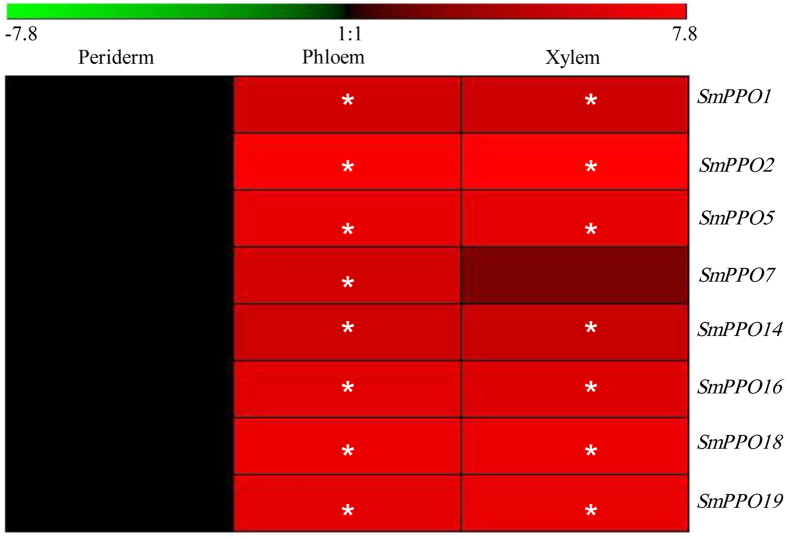
Expression of *SmPPOs* in periderm, phloem and xylem of *S. miltiorrhiza* roots. RNA-seq reads were mapped to the cloned ORFs of *SmPPOs*. Genes with RPKM value greater than 2 were analyzed for differential expression using Fisher’s exact test. *P* < 0.05 was considered as differentially expressed. *Indicates significant differential expression compared with the level in periderm.

*S. miltiorrhiza* roots are well-known materials of various TCMs. The pharmacologically active phenolic acid, lithospermic acid B, accumulates mainly in the phloem and xylem of *S. miltiorrhiza* roots^22^. In order to elucidate the role of *SmPPOs* in *S. miltiorrhiza* roots, transcriptome-wide analysis were carried out. RNA-seq reads from periderm, phloem and xylem of *S. miltiorrhiza* roots were downloaded from GenBank and then mapped to *SmPPOs* using SOAP2.0^22^,^23^. The results showed that 8 *SmPPOs* were expressed in *S. miltiorrhiza* roots with RPKM value greater than 2^24^. All of them exhibited higher expression levels in the phloem and xylem than the peridem (Fig. 5), indicating their potential in lithospermic acid B biosynthesis and metabolism.

### Responses of *SmPPOs* to MeJA treatments

**Figure 6:**
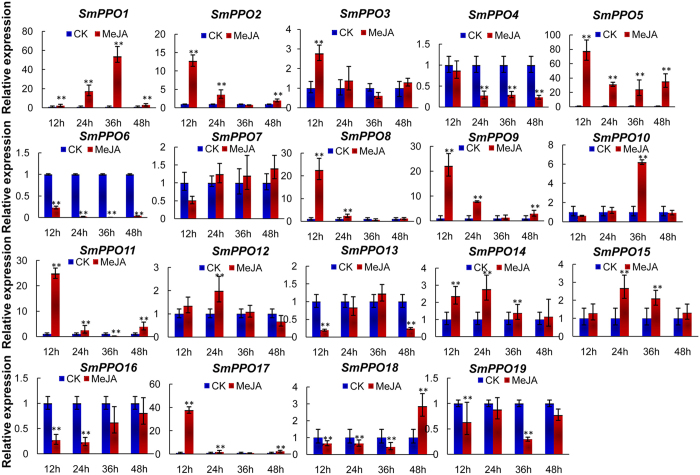
Quantitative RT-PCR analysis of *SmPPO* gene expression in *S. miltiorrhiza* leaves treated with MeJA. Fold changes of *SmPPOs* in leaves of *S. miltiorrhiza* plantlets treated with MeJA for 12, 24, 36 and 48 h are shown. The level of transcripts in leaves treated with carrier solution (CK) was arbitrarily set to 1 and the levels in leaves treated with MeJA were given relative to this. Mean values and standard deviations were obtained from three biological and three technical replicates. ANOVA (analysis of variance) was calculated using SPSS. *P* < 0.05(*) and *P* < 0.01(**) were considered statistically significant and extremely significant, respectively.

MeJA is an effective elicitor of tanshinone and phenolic acid production in *S. miltiorrhiza* and participates in plant response to stress^24^,^25^. To gain knowledge of *SmPPOs* in response to MeJA, the expression of *SmPPOs* in leaves of *S. miltiorrhiza* plantlets with or without MeJA treatment was performed using the qRT-PCR method. Significant expression level changes were observed for 18 of the 19 *SmPPOs* at one or more time-points a time-point of MeJA treatment (Fig. 6). A total of 11 *SmPPOs*, including *SmPPO1, SmPPO2, SmPPO3, SmPPO5, SmPPO8, SmPPO9, SmPPO10, SmPPO12, SmPPO14, SmPPO15* and *SmPPO17*, were significantly up-regulated at least a time-point of MeJA treatment. Among them, *SmPPO1* and *SmPPO5* were significantly up-regulated at all time-points of MeJA treatment. Five *SmPPOs*, including *SmPPO4, SmPPO6, SmPPO13, SmPPO16*, and *SmPPO19*, were significantly down-regulated at one or more time-points time-point of MeJA treatment. Among them, *SmPPO6* was down-regulated at all four time-points. The responses of *SmPPO11* and *SmPPO18* to MeJA treatment were fluctuated. *SmPPO11* was up-regulated at the time-points of 12-, 24- and 48-h-treatment, while down-regulated at the time-point of 36-h-treatment. *SmPPO18* was down-regulated at the time-points of 12-, 24- and 36-h-treatment, while up-regulated at the time-point of 48-h-treatment. No significant changes were observed for *SmPPO7* after MeJA treatment. The results suggest that the majority of *SmPPOs* are involved in response to MeJA treatment in *S. miltiorrhiza*.

### Yeast extract and Ag^+^-responsive *SmPPOs*

**Figure 7:**
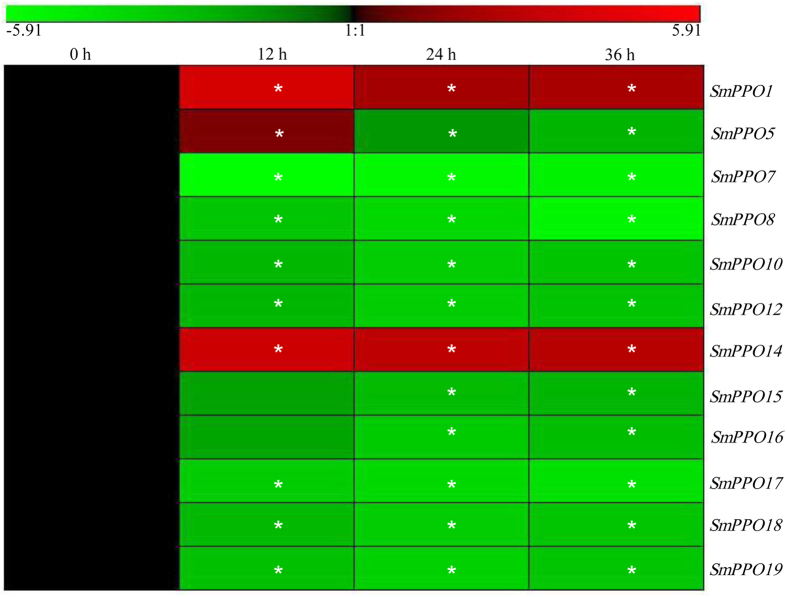
Responses of *SmPPO* genes to yeast extract and Ag^+^ treatment. RNA-seq reads were mapped to the cloned ORFs of *SmPPO* genes. Genes with RPKM value greater than 2 were analyzed for differential expression using Fisher’s exact test. *P* < 0.05 was considered as differentially expressed. *Indicates significant differential expression compared with the level in hairy roots without treatment.

It has been shown that PPOs are involved in plant response to stress^5^. In order to determine whether SmPPOs play a role in plant defense, we carried out a transcriptome-wide analysis of *SmPPO* expression in response to the treatment of yeast extract (100 μg/ml) and Ag^+^ (30 μM), a combination of biotic and abiotic stresses^24^. RNA-seq data of *S. miltiorrhiza* hairy roots treated with or without yeast extract (100 μg/ml) and Ag^+^ (30 μM) were downloaded from GenBank and then mapped to *SmPPOs* using the SOAP2.0 software^23^,^24^. Using a cutoff of RPKM value greater than 2^24^, a total of 12 *SmPPOs* were found to be expressed in hairy roots. Compared with the level in non-treated control, all of the 12 *SmPPOs* expressed in hairy roots were differentially expressed at all three time-points of yeast extract and Ag^+^ treatment (Fig. 7). *SmPPO5* was up-regulated at the time-point of 12-h-treatment, whereas down-regulated after 24- and 36-h-treatment. *SmPPO1* and *SmPPO14* were significantly up-regulated at all time points. The other 9 *SmPPOs* were significantly down-regulated at all time points (Fig. 7). It suggests that over 60% of *SmPPOs* are yeast extract and Ag^+^-responsive.

### *SmPPOs* are not regulated by miR1444 in *S. miltiorrhiza*

In *Salicaceae*, a subset of *PPOs* are regulated by a microRNA, termed miR1444^7^,^15^,^21^. This microRNA is a lineage-specific young microRNA^15^. MiR1444-mediated regulation of *PtPPOs* is significant in copper homeostasis and stress responses in *P. trichocarpa*^7^. In order to examine whether *SmPPOs* are regulated by miR1444, we analyzed published and our own high throughput sequencing data of small RNAs from roots, stems, leaves and flowers of *S. miltiorrhiza*^26^,^27^. No miR1444 sequence was found. We next analyzed the whole genome sequence of *S. miltiorrhiza*^28^. Consistently, no *MIR1444* precursor sequence was identified. Taken together, we conclude that miR1444 does not exist in *S. miltiorrhiza* and *SmPPOs* are not regulated by miR1444.

### Identification of Smi-miR12112 targeting 15 *SmPPOs* for cleavage in *S. miltiorrhiza*

**Figure 8:**
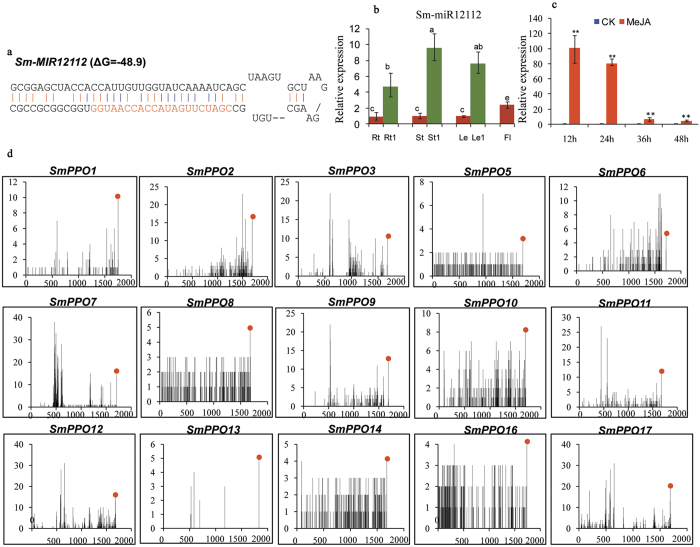
Smi-miR12112 targets *SmPPOs* for cleavage. (**a**) Predicted hairpin structures of Smi-miR12112. Mature miRNA sequences are indicated in red. The secondary structure of *Smi-miR12112* was predicted by the mfold program using the default parameters^30^. (**b**) Expression of Smi-miR12112 in *S. miltiorrhiza*. Relative expression of Smi-miR12112 was quantified in total RNA isolated from roots (Rt), stems (St), leaves (Le) and flowers (Fl) of 2-year-old, field-grown *S. miltiorrhiza* Bunge (line 993) and roots (Rt1), stems (St1) and leaves (Le1) of two-month-old plants cultivated *in vitro* by quantitative real-time RT-PCR and normalized to the level of 5.8S rRNA in the sample. (**c**) Expression of Smi-miR12112 in *S. miltiorrhiza* leaves treated with MeJA. Fold changes of *SmPPOs* in leaves of *S. miltiorrhiza* plantlets treated with MeJA for 12, 24, 36 and 48 h are shown. The level of transcripts in leaves treated with carrier solution (CK) was arbitrarily set to 1 and the levels in leaves treated with MeJA were given relative to this. Mean values and standard deviations were obtained from three biological and three technical replicates. ANOVA (analysis of variance) was calculated using SPSS. *P* < 0.01(**) were considered statistically extremely significant. (**d**) Validation of Smi-miR12112**-**directed cleavage using degradome analysis. X-axis shows the nucleotide (nt) position of targets and Y-axis shows the reads obtained by degradome sequencing. Each black spot represents a degradome fragment mapped to the target gene. The red spots indicate that the products are resulted from Smi-miR12112-directed cleavage.

In order to elucidate whether *SmPPOs* are regulated by other microRNAs, we searched *S. miltiorrhiza* small RNAs potentially targeting *SmPPOs* for cleavage using psRNATarget^29^. With the maximum expectation of 3.0 applied in the target search, a total of 54 small RNAs with sequence reads greater than four were identified. These small RNAs were aligned with the whole genome sequence of *S. miltiorrhiza*^28^. Secondary structures were predicted for the retrieved genomic DNA sequences using mfold^30^ as described previously^31^. The structures were manually checked and miRNAs were annotated by applying the criteria suggested by Meyers *et al*.^32^. As a result, we finally identified a miRNA stem-loop structure designated as *Smi-MIR12112* (Fig. 8a). Smi-miR12112 is a novel miRNA that has not been reported previously.

Using the poly(A) adaptor RT-PCR method^33^, we analyzed the expression of Smi-miR12112 in roots, stems, leaves and flowers of 2-year-old, field-grown *S. miltiorrhiza* Bunge (line 993) and roots, stems and leaves of two-month-old plants cultivated *in vitro*. Smi-miR12112 exhibited higher expression level in young roots, young stems and young leaves compared with the level in mature roots, mature stems, mature leaves and flowers (Fig. 8b). High level of expression in young tissues indicates that Smi-miR12112 plays more important roles in young tissues than mature ones. In order to determine whether Smi-miR12112 is transcriptionally regulated by MeJA, we analyzed the expression of Smi-miR12112 in leaves of *S. miltiorrhiza* plantlets with or without MeJA treatment using the qRT-PCR method. The results showed that the level of Smi-miR12112 was up-regulated at all four time-points (Fig. 8c). Differential response of Smi-miR12112 (Fig. 8c) and *SmPPOs* (Fig. 6) to MeJA treatment suggest the complexity of the miRNA and *SmPPO*-associated gene regulatory networks.

**Figure 9:**
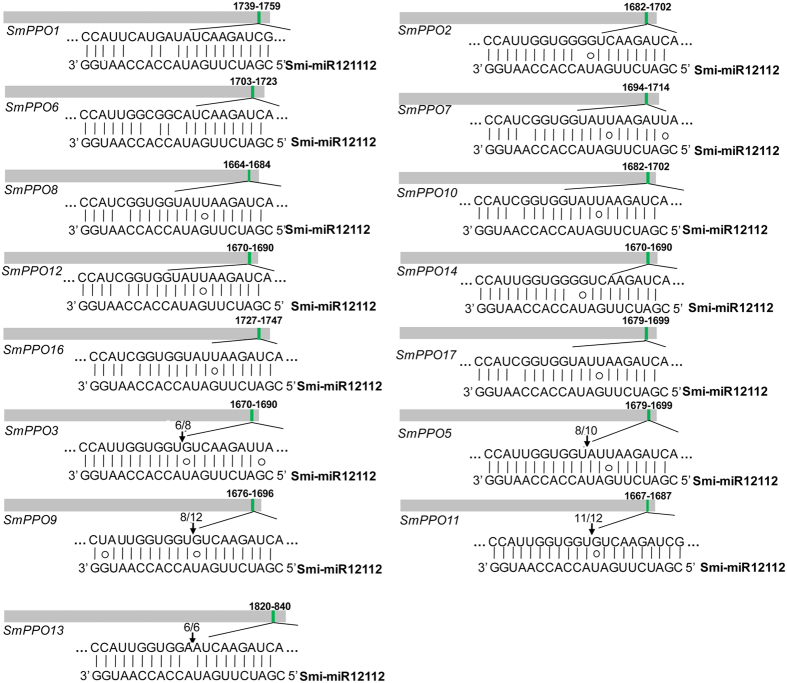
Locations of Smi-miR12112 complementary sequences in *SmPPOs*. Heavy grey lines represent ORFs. Smi-miR12112 complementary sites (green) with the nucleotide positions of *SmPPO* are indicated. The mRNA sequence of each complementary site from 5′ to 3′ and the mautre Smi-miR12112 sequence from 3′ to 5′ are shown in the expanded regions. Watson-Crick pairing (vertical dashes) and G:U wobble pairing (circles) are indicated. Vertical arrows indicate the 5′ termini of miRNA-guided cleavage products, as identified by 5′-RACE, with the frequency of clones shown.

Computational target prediction showed that 15 of the 19 identified *SmPPOs* contained a sequence near-perfectly complementary to Smi-miR12112 (Fig. 9). The sequence locates in a region encoding KFDV, a conserved domain of PPO proteins. Large proportion of *SmPPOs* having putative miRNA target sites could be due to deep conservation of sequence in the KFDV region. Plant mature miRNAs usually guide RNA-induced silencing complexes (RISCs) to cleave target mRNAs at the tenth complementary nucleotide from the 5′ end of the miRNA^34^. Based on this ruler, we analyzed our *S. miltiorrhiza* degradome sequencing data set for *SmPPO* cDNA fragments resulted from Smi-miR12112-mediated cleavage. The results confirmed the 15 *SmPPOs* to be targets of Smi-miR12112 (Fig. 8c). To further validate miRNA-mediated cleavage of predicted targets, we carried out rapid amplification of 5′ complementary DNA ends (5′-RACE). The results showed that five Sm*PPOs*, including *SmPPO3, SmPPO5, SmPPO9, SmPPO11* and *SmPPO13*, were indeed cleaved by Smi-miR12112 *in vivo* (Fig. 9). It verifies the results from computational prediction.

## Discussion

*PPOs* exist in land plants, fungi and some bacteria, such as *P. patens*^17^,^35^, *P. trichocarpa*^1^,^7^,^8^ potato^36^, tomato^37^, walnut^38^, eggplant^39^, sugarcane^17^, litchi^40^, *Vicia faba*^41^, and grapevine^42^. PPOs are encoded by a gene family with member numbers varied significantly among species^17^. The non-vascular moss, *P. patens*, contains thirteen *PPO* members^36^. Soybean has eleven. Sorghum (*Sorghum bicolor*) has eight. Maize (*Zea mays*) and purple false brome (*Brachypodium distachyon*) contain six. Fox millet (*Setaria italica*) has four. Rice contains two. Cucumber, cassava, castor bean and walnut have one^6^,^38^. No PPO was detected in *Arabidopsis, Brassica napus* and green algae (*Chlorella, Stigeoclonium, Microspora, Ulva* and *Spirogyra*)^17^. The lack of a *PPO* gene in various plant species points to the ecological or secondary metabolic functions for *PPOs*^16^. Consistently, the number of *PPO* genes in an organism is not directly related to the size of its genome. For instance, the *Selaginella moellendorffii* genome (~100 Mbp) is one of the smallest plant genomes known; however, the number of *PPO* genes, 11, in this species is relatively greater^16^. Analysis of the *P. trichocarpa* genome assembly v3.0 (http://www.phytozome.net/poplar.php#B) showed the existence of 15 full-length *PtPPO* genes. In this study, we identified a total of 19 full-length *PPO* genes in *S. miltiorrhiza*. It suggests that *S. miltiorrhiza* contains the largest *PPO* gene family in plant species with the whole genome sequence available.

It has been shown that land plant *PPO* genes originate in bacteria via an ancient horizontal gene transfer event^43^. The transferred *PPO* gene may expand through gene duplication or lost through chromosome rearrangement or fragment deletion during plant evolution, resulting in significant variation in *PPO* gene numbers in different plant species. The lack of *PPO* genes in *Arabidopsis* suggests that *PPO* genes may be lost through deletion and mutation during chromosome rearrangement. Consistently, phylogenetic and gene structure analysis indicated that *PPO* genes are relatively conserved across different species. For instance, most PPO proteins share similar domains and motifs across plants. The intron/exon structures of *PPO* genes are also largely conserved. The evolution history suggests that *PPO* genes from different organisms may share conserved molecular functions.

Recent studies implicate that PPOs are involved in the biosynthesis of phenolic acids. PPOs from *Portulaca grandiflora* and other plants in the Caryophyllales are able to hydroxylate tyrosine to 3, 4-dihydroxyphenylalanine (DOPA) and are considered to be one of the key enzymes for the biosynthesis of water-soluble pigment betalains, which replace the anthocyanins in flowers and fruits of most Caryophyllales plants^44^,^45^,^46^,^47^,^48^. A vacuole-localized PPO from snapdragon (*Antirrhinum majus*), named AmAS1, specifically catalyzes the formation of aurones from chalcones, a class of plant flavonoids responsible for the yellow coloration of flowers^17^. Larreatricin hydroxylase (LtLH), a typical PPO from creosote bush (*Larrea tridentate*), specifically hydroxylates (+)-larreatricin to (+)-3′-hydroxylarreatricin and is thought to play a central role in the biosynthesis of the creosote bush 8–8′ linked lignans^49^. In walnut, PPO is involved in the phenylpropanoid pathway and the tyramine pathway and acts as an indirect regulator of cell death^38^. Phenolic acids are a group of bioactive compounds in *S. miltiorrhiza*. The SmPPOs invovled in phenolic acid biosynthesis are currently unknown. Further analysis of SmPPO functions through genetic transformation will definitely shed lights on the mechanism of phenolic acid biosynthesis in *S. miltiorrhiza*. In addition to be involved in phenolic acid biosynthesis, the diversified expression patterns indicate that *SmPPOs* may also play significant roles in other physiological processes. Evidence obtained in this study includes that the expression of *SmPPOs* exhibits apparent tissue specificity (Fig. 4), and the majority of *SmPPOs* are responsive to MeJA treatment (Fig. 6) and yeast extract and Ag^+^ treatment (Fig. 7).

miRNAs are a class of small endogenous non-coding RNAs with size about 21 nucleotides. They play vital roles in multiple developmental and physiological processes in various organisms through sequence-specific regulation of target genes at the transcriptional or post-transcriptional level^50^. MiRNA-mediated posttranscriptional regulation is important for the function of a subset of *PPOs* in *P. trichocarpa* and grapevine^15^,^51^. In *P. trichocarpa*, at least 13 *PtPPOs* are regulated by miR1444^7^. In grapevine, *VvPPO* is regulated by miR058^51^. Although miRNAs have been studied extensively in the past several years, only few documents have been reported for miRNAs in *S. miltiorrhiza*^26^,^27^,^52^,^53^,^54^,^55^. The functions of most *S. miltiorrhiza* miRNAs are still unknown. Analysis of small RNA data revealed the loss of miR1444 in *S. miltiorrhiza*. Instead, a novel *PPO*-targeting miRNA, designated as Smi-miR12112, exists in *S. miltiorrhiza*. It targets to 15 of the 19 identified *SmPPOs* in a region encoding the conserved KFDV domain. The target site of Smi-miR12112 is different from those of Pt-miR1444s and Vv-miR058, which locate in the regions encoding the conserved CuB domain and the thylakoid transfer domain, respectively. Since miRNA target sites generally occur outside of family-defining domains^56^, the origination and evolution of PPO conserved domain-targeting miRNAs may be under strong negative selection. It suggests the significance of miRNA-mediated posttranscriptional regulation of PPOs in plants.

## Materials and Methods

### Plant materials and treatments

*Salvia miltiorrhiza* Bunge (line 99–3) with whole genome sequence available was grown in a field nursery at the Institute of Medicinal Plant Development. Flowers, leaves, stems and roots were harvested from 2-year-old plants in June when *S. miltiorrhiza* is booming, and stored in liquid nitrogen until use. Leaves were treated with MeJA (200 μM) for 12 h, 24 h, 36 h, and 48 h and collected as described in a previous study^57^. Plantlets treated with carrier solution were used as controls. Three independent biological replicates were carried out for each experiment.

### Gene prediction and cDNA cloning

The amino acid sequences of 15 *P. trichocarpa* PPOs (PtPPOs) were downloaded from the *P. trichocarpa* genome assembly v3.0 (http://www.phytozome.net/poplar.php#B). To obtain sequencing data for all of the *SmPPO* genes, tBLASTn searches were performed on the genome database of *S. miltiorrhiza* line 99-3 (http://www.ndctcm.org/)^28^. An e-value cut-off of e^−10^ was applied. Gene model prediction was carried out for the retrieved genomic DNA sequences on the GENSCAN web server (http://genes.mit.edu/GENSCAN.html). The predicted gene models were examined and comparatively analyzed with the other genome database of *S. miltiorrhiza* (http://www.herbal-genome.cn)^58^.

To clone the full-length *SmPPO* cDNAs, rapid amplification of 5′ (5′-RACE) and 3′ (3′-RACE) cDNAs ends was performed using the SMART^TM^ RACE cDNA amplification kit (TaKaRa Bio, Otsu, Japan). The nesting and the nested PCR amplification was performed on cDNA reverse-transcribed from total RNA using primers listed in Supplementary Tables S1 and S2. Full-length coding sequences were amplified by PCR using a combination of gene-specific forward primers and reverse primers (Supplementary Table S3). PCR products were purified, cloned and sequenced.

### Bioinformatic analysis and phylogenetic tree construction

The theoretical isoelectric point (p*I*) and molecular weight (Mw) were analyzed using the Compute p*I*/Mw tool on the ExPASy server (http://web.expasy.org/compute_pi/)^59^. The conserved domain of SmPPO proteins was searched against the Conserved Domain Database (CDD, http://www.ncbi.nlm.nih.gov/Structure/cdd/wrpsb.cgi). The expected e-value threshold of 1.0 and the maximum size of hits to be 500 amino acids were applied^60^. Sequence logos were created on the WebLogo server (http://weblogo.berkeley.edu/logo.cgi)^61^.

Protein sequences of PPOs from *P. trichocarpa* (PtPPOs), *Glycine max* (GmPPOs), *Oryza sativa* (OsPPOs), *Zea mays* (ZmPPOs) and *Physcomitrella patens* (PpPPOs) were downloaded from Phytozome (http://phytozome.jgi.doe.gov/pz/portal.html) and NCBI (http://www.ncbi.nlm.nih.gov/protein/) (Supplementary Table S4). The phylogenetic tree was constructed using MEGA 7.0, with 1000 bootstrap replicates^62^.

### Paralog identification and synteny analysis

Paralog groups were identified using BLASTP with the following criteria applied. It includes *E* value ≤10–40, cumulative identity percentage (CIP) ≥60%, and cumulative alignment length percentage (CALP) ≥70%. The paralog groups were recognized as homologous *SmPPO* gene groups among all of the *SmPPO* genes identified in the *S. miltiorrhiza* genome. cDNA sequences of each paralogous group and orthologous group were subjected to multiple sequence alignments. The numbers of nonsynonymous substitutions per nonsynonymous site (*Ka*) and synonymous substitutions per synonymous site (*Ks*) were calculated using MEGA 7.0^62^.

### Quantitative real-time reverse transcription-PCR (qRT-PCR)

The first cDNA strand was reversely transcribed using SuperScript Ш Reverse Transcriptase (Invitrogen, Carlsbad, CA, USA). qRT-PCR analysis of *SmPPOs* in flowers, leaves, stems and roots of 2-year-old plants and in plantlets treated with MeJA was carried out as described previously^57^. Gene-specific primers were designed using the tool of IDT designing primers (http://www.idtdna.com/scitools/Applications/RealTimePCR/) (Supplementary Table S5). The length of amplicons was between 80 bp and 200 bp. *SmUBQ10* was used as an internal control as previously described^54^,^57^. The expression of Smi-miR12112 was analyzed using the poly(A) adaptor RT-PCR method as described previously^33^. Real-time PCR was performed using 5′-CGATCTTGATACCACCAATGG-3′ as the forward primers and 5′-GCGAGCACAGAATTAATACGAC-3′ as the reverse primer. 5.8S rRNA gene was selected as a reference^7^. Gene expression data from three biological replicates was standardized as described previously^63^. ANOVA (analysis of variance) was calculated using SPSS (Version 19.0, IBM, USA). *P* < 0.05 was considered statistically significant, and *P* < 0.01 was considered extremely significant.

### Analysis of *SmPPOs* expression using RNA-seq data

RNA-seq data from various *S. miltiorrhiza* tissues was downloaded from GenBank. The tissues include periderm, phloem and xylem of roots (SRR1640458), hairy roots, and hairy roots treated with yeast extract (100 μg/ml) and Ag^+^ (30 μM) (SRR924662)^22^,^24^. RNA-seq reads were mapped to *SmPPOs* using SOAP2.0^23^ and analyzed as described previously^57^. *SmPPOs* with the RPKM value greater than 2 were analyzed for differential expression using Fisher’s exact test. *P* < 0.05 was considered as differentially expressed.

### Identification of *S. miltiorrhiza* miRNAs with perfect or near-perfect complementarity to *SmPPOs*

*S. miltiorrhiza* small RNAs with the potential to target *SmPPOs* for cleavage were predicted using psRNATarget^29^. The nineteen *SmPPO* genes identified were used as target transcript candidates. The maximum expectations of 3.0 and the target accessibility-allowed maximum energy to unpair the target site of 25.0 were applied. The identified small RNAs were aligned with the genome of *S. miltiorrhiza*^27^. Secondary structures of *S. miltiorrhiza* genomic DNA sequences with small RNAs aligned were predicted using mfold^30^. In each case, the lowest energy structure was analyzed as described previously^30^.

### Degradome and experimental verification of miRNA-directed cleavage of *SmPPOs*

Degradome analysis of miRNA-targeted *SmPPOs* was carried out using SOAP2.0^23^. To map miRNA cleavage sites in *SmPPO* targets, the modified RNA ligase-mediated rapid amplification of 5′ cDNAs method (5′RLM-RACE) was performed using the SMART^TM^ RACE cDNA amplification kit (TaKaRa Bio, Otsu, Japan). The nesting and the nested primers used in this experiment are listed in Supplementary Table S6. Nesting PCR amplification was performed under the following program of touchdown PCR: 94 °C for 5 min, 5 cycles of amplification 94 °C for 30 s and 72 °C for 3 min, 5 cycles of amplification at 94 °C for 30 s, 70 °C for 30 s and 72 °C for 3 min, 25 cycles of amplification at 94 °C for 30 s, 56 °C for 30 s and 72 °C for 3 min, followed by a final extension at 72 °C for 10 min. Nested PCR amplification was carried out under the following conditions: 94 °C for 5 min, 25 cycles of amplification at 94 °C for 30 s, 56 °C for 30 s and 72 °C for 3 min, followed by a final extension at 72 °C for 10 min. PCR products were purified, cloned and sequenced.

## Additional Information

**How to cite this article:** Li, C. *et al*. Characterization of the polyphenol oxidase gene family reveals a novel microRNA involved in posttranscriptional regulation of *PPOs* in *Salvia miltiorrhiza. Sci. Rep.* **7**, 44622; doi: 10.1038/srep44622 (2017).

**Publisher's note:** Springer Nature remains neutral with regard to jurisdictional claims in published maps and institutional affiliations.

## Supplementary Material

Supplementary Information

## Figures and Tables

**Table 1:** Sequence features of *SmPPOs*.

Gene name	Accession no.	ORF (bp)	AA len	p*I*	Mw (Da)
*SmPPO1*	KX458045	1773	590	6.56	66010.64
*SmPPO2*	KX458046	1713	570	6.06	64457.11
*SmPPO3*	KX458047	1701	566	6.49	63976.95
*SmPPO4*	KX458048	1695	564	6.41	64061.26
*SmPPO5*	KX458049	1716	571	6.55	63827.14
*SmPPO6*	KX458050	1746	581	6.25	65018.72
*SmPPO7*	KX458051	1734	577	5.90	64231.44
*SmPPO8*	KX458052	1704	567	6.42	64143.58
*SmPPO9*	KX458053	1716	571	8.41	64190.41
*SmPPO10*	KX458054	1728	575	5.23	64820.16
*SmPPO11*	KX458055	1704	567	5.66	63326.35
*SmPPO12*	KX458056	1727	580	5.69	65575.18
*SmPPO13*	KX458057	1878	625	8.60	70294.13
*SmPPO14*	KX458058	1701	566	6.39	63573.64
*SmPPO15*	KX458059	1657	551	5.91	61922.95
*SmPPO16*	KX458060	1767	588	5.61	65957.20
*SmPPO17*	KX458061	1719	572	5.77	64851.20
*SmPPO18*	KX458062	1692	563	5.28	63775.88
*SmPPO19*	KX458063	1710	569	6.30	64738.34

ORF, open reading frame; AA len, the number of amino acid residues; p*I*, theoretical isoelectric point; Mw, molecular weight.

**Table 2:** Paralogous groups of *SmPPOs* in *S. miltiorrhiza*.

Paralogous group	Gene name
1	*SmPPO10, SmPPO15, SmPPO16*
2	*SmPPO12, SmPPO18, SmPPO19*
3	*SmPPO5, SmPPO7*
4	*SmPPO8, SmPPO11, SmPPO17*
5	*SmPPO2, SmPPO3, SmPPO9, SmPPO14*
6	*SmPPO6, SmPPO13*

**Table 3:** TargetP prediction of SmPPO localization.

Name	cTP	mTP	SP	other	Loc	RC
SmPPO1	0.966	0.022	0.028	0.084	C	1
SmPPO2	0.911	0.162	0.021	0.029	C	2
SmPPO3	0.808	0.136	0.022	0.091	C	2
SmPPO4	0.727	0.200	0.080	0.046	C	3
SmPPO5	0.975	0.030	0.008	0.060	C	1
SmPPO6	0.969	0.067	0.004	0.076	C	1
SmPPO7	0.977	0.018	0.009	0.077	C	1
SmPPO8	0.966	0.126	0.019	0.023	C	1
SmPPO9	0.765	0.099	0.032	0.078	C	2
SmPPO10	0.977	0.078	0.014	0.028	C	1
SmPPO11	0.966	0.126	0.019	0.023	C	1
SmPPO12	0.979	0.118	0.007	0.021	C	1
SmPPO13	0.929	0.132	0.035	0.009	C	2
SmPPO14	0.699	0.127	0.046	0.076	C	3
SmPPO15	0.954	0.096	0.007	0.065	C	1
SmPPO16	0.979	0.053	0.022	0.024	C	1
SmPPO17	0.979	0.088	0.118	0.007	C	1
SmPPO18	0.980	0.039	0.021	0.077	C	1
SmPPO1	0.966	0.022	0.028	0.084	C	1

cTP, chloroplast transit peptide; mTP, mitochondrial targeting peptide; SP, signal peptide; Loc, protein localization; C, chloroplast; RC, reliability class from 1 to 5, where 1 indicates the strongest prediction.
