# Supplementary material for: Characterization of the polyphenol oxidase gene family reveals a novel microRNA involved in posttranscriptional regulation of PPOs in Salvia miltiorrhiza
Source: Sci Rep. 2017 Mar 17;7:44622. doi: 10.1038/srep44622 (PMC5356337; doi:10.1038/srep44622)
Supplement: Supplementary Information [file srep44622-s1.pdf]

Supplementary information for Scientific Reports

Characterization of the polyphenol oxidase gene family reveals a novel microRNA involved in posttranscriptional regulation of *PPOs* in *Salvia miltiorrhiza*

Caili Li#, Dongqiao Li#, Jiang Li, Fenjuan Shao, Shanfa Lu*

Institute of Medicinal Plant Development, Chinese Academy of Medical Sciences & Peking Union Medical College, No.151, Malianwa North Road, Haidian District, Beijing 100193, China.

Supplementary Table S1 Primers used for 5'-RACE of *SmPPOs*.

Gene name	Primer sequence (5'-3')
SmPPO13	Nesting: TGGATTCCGGGTCATGCCGAGATCC<br>Nested: GTCAGACATCGGTGGGCAGCAGTTCATG
SmPPO14	Nesting: AGATCCGTGGGAGCAGGCGGGAGAT<br>Nested: TTGCATGAAACTGCGCGGGTCATCG
SmPPO15	Nesting: CGTACGTATTCCGGTGTCAGCCTGTGCG<br>Nested: TGCGATTGAGTGGGTTGCGGTTGCAG
SmPPO16	Nesting: CCTGTTCGTAGCGGCCGGTACAGTAAGC<br>Nested: AGCCGCCCTCCGACAATCCTCCCTT
SmPPO17	Nesting: TCTTCTACGCACATCACGCCAACG<br>Nested: TCTTTCCTTACCCAGGGCCTCAATC
SmPPO18	Nesting: CCCGACGACTTCAACAACGCCTCC<br>Nested: GAGGATTGTCGGAGGGCGGCTGTCT
SmPPO19	Nesting: ATCTCTAGACGTCAACTGCTGCCCG<br>Nested: CGCTTCCGATTTTGCCTCCTGACCT

Supplementary Table S2 Primers used for 3'-RACE of *SmPPOs*.

Gene name	Primer sequence (5'-3')
SmPPO17	Nesting: CACGGAGTTTCGCTTTCTTCATAC<br>Nested: ACTTCGTCGGCTTTGCCTTTGGTTG
SmPPO18	Nesting: ACTTCGTCGGCTTTGCCTTTGGT<br>Nested: CGCTTTCCGCAGTCCTAACCTTAT
SmPPO19	Nesting: TGGCGAGAACTTTGGCATTGACC<br>Nested: CGTCGGCTTTGCCTTTGGTTGGT

Supplementary Table S3 Primers used for the PCR-amplification of coding sequences of *SmPPOs*

Gene name	Primer sequence (5'-3')
SmPPO1	Forward: ATGGCTTCGTCGATTCTCTCACAT<br>Reverse: GTCATCAAGCTCGATCTTGATATCATG
SmPPO2	Forward: ATGTCATCTGTTCCTCTTTCCACCG<br>Reverse: CCACCAATGGTAATATCTTCTCCTCCA
SmPPO3	Forward: ATGGCATTTCCTCCACTTTCCTC<br>Reverse: TGACACCACCAATGGTAATGTCTTC
SmPPO4	Forward: ATGGCCTCTCTTCAATCTTCATGC<br>Reverse: CGCGACTTAACAATTTTGATACCA
SmPPO5	Forward: ATGGCTTCCCTTCAATCTTCTTTCTC<br>Reverse: GCGGGATGATCTTAATACCACCAAT
SmPPO6	Forward: ATGGCTTCCCTCTACCTTCATTGCA<br>Reverse: AGCTTTAGGTGGATTCTCAATGATCTTGA
SmPPO7	Forward: ATGGCTTCTCTTCCATCTTCACTCA<br>Reverse: AGCAGCAACACGCGGGATAATCT
SmPPO8	Forward: ATGGCTTCCCTTCAATCTTCATTC<br>Reverse: ACACGCGGGATGATCTTAATACC
SmPPO9	Forward: ATGGCTTCTTCCCTTCAGTCCTTAAG<br>Reverse: TTTACGCTGCGCTGGGATGA
SmPPO10	Forward: ATGGCTTCCCTTCAATCTTCATTCAC<br>Reverse: TCAAGCGGTAACACGCGGGAT
SmPPO11	Forward: ATGGCTTCTTCCCTTATTGCTTCATG<br>Reverse: ACGCTGCGCTGGGACGATCTT
SmPPO12	Forward: ATGGCTTCCCTTCAATCTTCATTCA<br>Reverse: AGCGGTAACACGCGGGATGATC
SmPPO13	Forward: ATGGCTTCCCTCTACCTTCATTGC<br>Reverse: TCAAGATCTTCGGGGTGGAGAAG
SmPPO14	Forward: ATGGCATCACTTCCGTTTTCCAC<br>Reverse: TTAAACGTAGATGATCTTGACCCCAC
SmPPO15	Forward: ATGGCTTCCCTTCAATCTTCATTC<br>Reverse: CACGCGGGATGATCTTAATACCA
SmPPO16	Forward: ATGGGGCACCAAACCATGGC<br>Reverse: TCAAGCGGTAACACGAGGAATGATC
SmPPO17	Forward: ATGCTCCTCGGTCTTGGAGGGC<br>Reverse: TCAAACGGGAACACGCGGGATG
SmPPO18	Forward: ATGGCGAAGATGAGGGAGCTG<br>Reverse: TCAAGCGGTAACACGCGGGATG
SmPPO19	Forward: ATGGCTTCCCTTCAATCTTC<br>Reverse: TCAAGCAGTAACACGCGGGATG

Supplementary Table S4 Gene names of *PPOs*.

**Gene name**	**Accession no.**
Populus trichocarpa	
PtPPO1	Potri.011G108300
PtPPO2	Potri.001G387900
PtPPO3	Potri.011G047300
PtPPO4	Potri.T062100
PtPPO5	Potri.001G388600
PtPPO6	Potri.001G388400
PtPPO7	Potri.001G388300
PtPPO8	Potri.T061900
PtPPO9	Potri.001G388100
PtPPO10	Potri.011G108200
PtPPO11	Potri.001G388900
PtPPO12	Potri.001G388800
PtPPO13	Potri.004G156500
PtPPO14	Potri.001G388200
PtPPO15	Potri.T062200
PtPPO16	Potri.001G388000
Glycine max	
GmPPO1	Glyma06g42170.1
GmPPO2	Glyma07g31270.1
GmPPO3	Glyma07g31280.1
GmPPO4	Glyma07g31300.1
GmPPO5	Glyma07g31310.1
GmPPO6	Glyma13g25150.1
GmPPO7	Glyma13g25180.1
GmPPO8	Glyma13g25260.1
GmPPO9	Glyma13g31590.1
GmPPO10	Glyma15g07710.1
GmPPO11	Glyma18g45900.1
Zea mays	
ZmPPO1	GRMZM2G108103_T01
ZmPPO2	GRMZM2G121605_T01
ZmPPO3	AC233851.1_FGT017
ZmPPO4	GRMZM2G319062_T01
ZmPPO5	GRMZM2G137909_T01
ZmPPO6	AC209206.3_FGT014
Oryza sativa	
OsaPPO1	LOC_Os01g58100.1
OsaPPO2	LOC_Os04g53300.1
Phycomitrella patens	
PpPPO1	AAX69084.1
PpPPO2	XP_001770109.1

PpPPO3	XP_001785624.1
PpPPO4	XP_001752158.1
PpPPO5	XP_001773770.1
PpPPO6	XP_001785949.1
PpPPO7	XP_001767174.1
PpPPO8	XP_001766337.1
PpPPO9	XP_001766259.1
PpPPO10	XP_001766553.1
PpPPO11	XP_001755077.1
PpPPO12	XP_001785245.1

Supplementary Table S5 Primers used for quantitative real-time RT-PCR of *SmPPOs*

Gene name	Primer sequence (5'-3')
SmPPO1	Forward: GAAGAGGAGCAAGAAGGAGAAG<br>Reverse: TCGTCCTCGTCGTTGATGTA
SmPPO2	Forward: TCGTGAGGTTTGAGGTGTTC<br>Reverse: AGCCTGATCTTGCTCTTCAC
SmPPO3	Forward: GGTAAGGGTGTTGGTGGATAG<br>Reverse: TATTTGGCGGTGTCGATCTC
SmPPO4	Forward: GCCTTCTACTCCCATCACTCTA<br>Reverse: CGTTGAGGTAGTCAGGTTTGTT
SmPPO5	Forward: CGGTCAATCTCAAGGACATCT<br>Reverse: CCTTTGGTTGGTTTGGACAC
SmPPO6	Forward: GCATGTGGACTCTATGGAAGG<br>Reverse: GAAAGAGGCGTTGAGGTAGTC
SmPPO7	Forward: CACAGTGTTCCCTCTCAAACT<br>Reverse: CCTCACGAGCTTGCTGTTAT
SmPPO8	Forward: CAAAGTGTTCCCTCTCACTCTC<br>Reverse: AACTCACCAGCTTGGAACTATC
SmPPO9	Forward: CTGAAGAAGGCGGTTAGAGTTT<br>Reverse: TTGGTGCAATCGGTCTCTATG
SmPPO10	Forward: GGTGCATATCGAAGAGTGGAT<br>Reverse: GAGAGTGAGAGGGAACAGATTG
SmPPO11	Forward: GGAGCTCTACGAGAACATTGAC<br>Reverse: GACGATCTTGACACCACCAA
SmPPO12	Forward: CAATCTGTTCCCTCTCACTCTC<br>Reverse: ACCTCACGACCTTCAAACTATC
SmPPO13	Forward: TGTGGACTCTGTGGCAATC<br>Reverse: GCGTCCTCGTCGTAGAATAAG
SmPPO14	Forward: CGACTACGGCTAAGGCAATTA<br>Reverse: CTCCTTCTCCTTCTTGCTTCTC

SmPPO15	Forward: AACTCCATGGATGGCTTACAG<br>Reverse: ACAGTGTTGGTGAGAGTGATG
SmPPO16	Forward: CGTGTATGAACACAGCGAAAC<br>Reverse: GAGAGTGAGAGGGAACAGATTG
SmPPO17	Forward: CAATGTGTTCCCTCTCACTCTC<br>Reverse: AACTCACCAGCTTGGAACTATC
SmPPO18	Forward: TCGTGAGGTTCGATGTGTTC<br>Reverse: CTTTCCGCAGTCCTAACCTTAT
SmPPO19	Forward: CCTCTCACTCTCACCAAGACTA<br>Reverse: CCTCACGATCTTGGACCTTTC

Supplementary Table S6 Primers used for analysis of Smi-miR12112-directed cleavage of *SmPPOs*

Gene name	Primer sequence (5'-3')
SmPPO3	Nesting: GCAGCAAGCAGTACAAGTAGTG<br>Nested: GTGTAGACGATGATTCACAGAAG
SmPPO5	Nesting: GTCGACAAATCTCAAGCAACATGGAG<br>Nested: GAAACATTGGCGATGGATGCAG
SmPPO9	Nesting: CGGTTAACCGAACCATAAACCGG<br>Nested: GATATGTAGATTTTATTGTGGAGC
SmPPO11	Nesting: GCTTCCCCTCTCTCACACACACAC<br>Nested: CATTGGAGGAATTGGAACATGCG
SmPPO13	Nesting: GGTCTGCCTCCATGGAACGATTGTC<br>Nested: GGAGAAATGCTAGCGTAGGGC

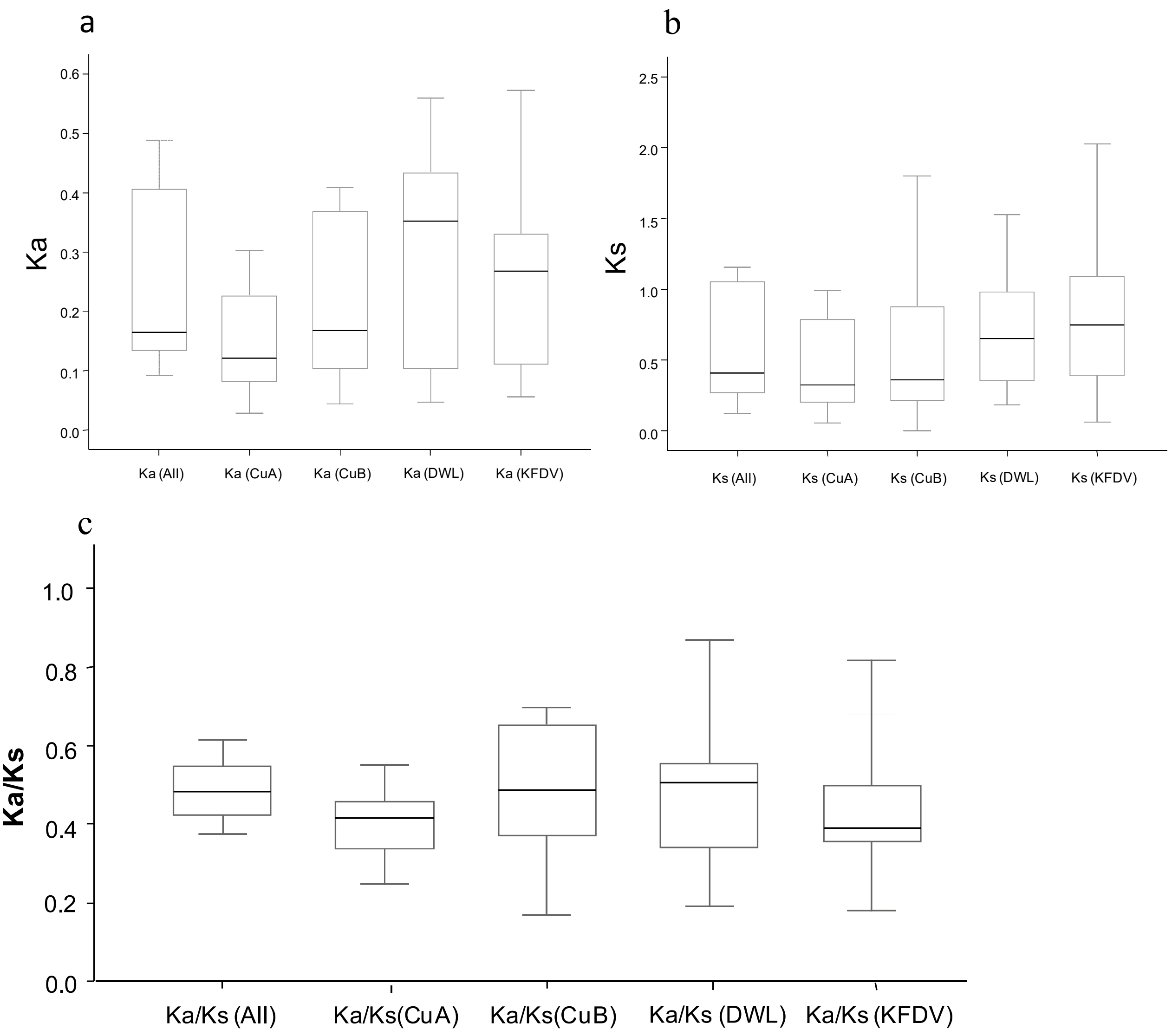

Supplementary Figure S1 Divergence of different classes in SmPPOs. (a) the Ka values of different classes in SmPPOs; (b) the Ks values of different classes in SmPPOs; (c) the Ka/Ks ratios of different classes in SmPPOs.
